# Hardy-Weinberg Equilibrium in the Large Scale Genomic Sequencing Era

**DOI:** 10.3389/fgene.2020.00210

**Published:** 2020-03-13

**Authors:** Nikita Abramovs, Andrew Brass, May Tassabehji

**Affiliations:** ^1^School of Computer Science, University of Manchester, Manchester, United Kingdom; ^2^Faculty of Biology, Medicine and Health, School of Biological Sciences, University of Manchester, Manchester, United Kingdom; ^3^Faculty of Biology, Medicine and Health, School of Health Sciences, University of Manchester, Manchester, United Kingdom; ^4^Manchester Centre for Genomic Medicine, St Mary’s Hospital, Manchester Academic Health Sciences Centre (MAHSC), Manchester, United Kingdom

**Keywords:** Hardy-Weinberg Equilibrium, heterozygote advantage, gnomAD, association studies in genetics, recessive inheritance

## Abstract

Hardy-Weinberg Equilibrium (HWE) is used to estimate the number of homozygous and heterozygous variant carriers based on its allele frequency in populations that are not evolving. Deviations from HWE in large population databases have been used to detect genotyping errors, which can result in extreme heterozygote excess (HetExc). However, HetExc might also be a sign of natural selection since recessive disease causing variants should occur less frequently in a homozygous state in the population, but may reach high allele frequency in a heterozygous state, especially if they are advantageous. We developed a filtering strategy to detect these variants and applied it on genome data from 137,842 individuals. The main limitations of this approach were quality of genotype calls and insufficient population sizes, whereas population structure and inbreeding can reduce sensitivity, but not precision, in certain populations. Nevertheless, we identified 161 HetExc variants in 149 genes, most of which were specific to African/African American populations (∼79.5%). Although the majority of them were not associated with known diseases, or were classified as clinically “benign,” they were enriched in genes associated with autosomal recessive diseases. The resulting dataset also contained two known recessive disease causing variants with evidence of heterozygote advantage in the sickle-cell anemia *(HBB)* and cystic fibrosis *(CFTR)*. Finally, we provide supporting *in silico* evidence of a novel heterozygote advantageous variant in the chromodomain helicase DNA binding protein 6 gene (*CHD6*; involved in influenza virus replication). We anticipate that our approach will aid the detection of rare recessive disease causing variants in the future.

## Introduction

The Hardy-Weinberg Equilibrium (HWE) is an important fundamental principal of population genetics, which states that “genotype frequencies in a population remain constant between generations in the absence of disturbance by outside factors” (Edwards, 2008). According to HWE, for a locus with two alleles *A* and *a* with corresponding frequencies p and q, three genotypes are possible *AA*, *Aa*, and *aa* with expected frequencies p^2^, 2pq, q^2^, respectively (Graffelman et al., 2017). However, various factors, including mutation, natural selection, non-random mating, genetic drift, and gene flow can cause deviations from HWE (Graffelman et al., 2017). Positive and negative assertive mating might result in deviations from HWE due to heterozygote deficiency or excess respectively, although the latter is more rarely observed in humans (Thiessen and Gregg, 1980). Non-random mating due to geographical location might be a common cause of deviations from HWE due to heterozygous deficiency in large populations of different ethnicities (Garnier-Géré and Chikhi, 2013). If a population consists of several subpopulations and individuals randomly mate within, but not between subpopulations, then homozygous alleles in the overall population will be observed more frequently than expected by HWE (“Wahlund effect”) (Sinnock, 1975). A technical cause of deviations from HWE, sometimes observed in population studies, is sequencing errors (Chen et al., 2017; Graffelman et al., 2017). Previous studies found that variants deviated from HWE mainly due to heterozygote excess (60–69% of the cases) (Chen et al., 2017; Graffelman et al., 2017) and deviations were 11 times more frequently observed in unstable genomic regions such as segmental duplications and simple tandem repeats (Graffelman et al., 2017), that are prone to sequencing errors. These issues were addressed in the Genome Aggregation Database (gnomAD; release v2.1.1) (Karczewski et al., 2019a), currently the largest publicly available population variant database (137,842 predominantly healthy individuals from seven ethnic populations). Variants with extreme heterozygote excess in the database were excluded by gnomAD, whereas those located in repeat regions were marked as dubious.

A factor causing deviations from HWE that has not been investigated on a large scale, is natural selection. Although individuals with known severe pediatric diseases were excluded from gnomAD (Karczewski et al., 2019a), some disease causing variants persisted (Tarailo-Graovac et al., 2017). For example, the African specific (∼91% of the carriers) *HBB* c.20A > T (rs334) variant, is a known recessive pathogenic variant which causes sickle-cell disease (MIM:603903) (Ashley-Koch et al., 2000), but it is present in four African individuals (who could have sickle-cell disease) in a homozygous state in gnomAD. Moreover, this variant is present in a heterozygous state in ∼9% (1,113/12,482) of African individuals (i.e., unaffected carriers), which is significantly more (∼2.5 times) than the expected number (∼439 individuals) according to HWE (*P* = 1.38E-07), for that number of homozygous individuals. The presence of a recessive disease causing variant at a high frequency in populations may also due to overdominant selection, i.e., a heterozygous variant provides some advantage to carriers (Withrock et al., 2015), as is the case for *HBB* c.20A > T, which provides carriers protection from malaria (MIM:611162) (Allison, 1954). This example illustrates that variants deviating from HWE due to heterozygote excess may also be recessive disease causing and possibly heterozygote advantageous. Here we developed a variant filtering strategy to detect novel potential disease causing variants that might deviate from HWE due to natural selection, and applied it to population data from gnomAD.

## Materials and Methods

### Collecting Gene and Variant Datasets

The gene dataset, with disease phenotype and inheritance data from Online Mendelian Inheritance in Man (OMIM) database (Hamosh et al., 2005), was obtained from Gene Discovery Informatics Toolkit (GDIT) (Dawes et al., 2019) and consisted of 19,196 protein coding genes. Population variant data with clinical annotation (ClinVar; Landrum et al., 2016) was obtained from gnomAD (Karczewski et al., 2019a) via API^1^. The database consisted of 137,842 individuals from seven populations: Non-Finnish European (NFE, *n* = 64,603), Latino/Admixed American (AMR, *n* = 17,720), South Asian (SAS, *n* = 15,308), Finnish (FIN, *n* = 12,562), African/African American (AFR, *n* = 12,487), East Asian (EAS, *n* = 9,977), and Ashkenazi Jewish (ASJ, *n* = 5,185) (Karczewski et al., 2019a). The initial variant dataset consisted of more than 17 million unique variants in 18,214 genes whose names in the GDIT dataset were found in gnomAD.

### Filtering Initial Variant Dataset

Variants which satisfied the following criteria were selected for initial analysis of deviations from HWE: (i) Variant is located in the canonical transcript [as defined in gnomAD who used GENCODE (Frankish et al., 2019) v19 annotation]; (ii) Variant is located on an autosomal chromosome; (iii) Variant is protein coding, i.e., has one of the following Variant Effect Predictor (VEP) (McLaren et al., 2016) version 85 consequences: *“transcript_ablation,” “splice_acceptor_variant,” “splice_donor_variant,” “stop_gained,” “frameshift_variant,” “stop_lost,” “start_lost,” “transcript_amplification,” “inframe_insertion,” “inframe_deletion,” “missense_variant,” “protein_altering_variant,” “splice_region_variant,” “incomplete_terminal_codon_variant,” “start_retained_variant,” “stop_retained_variant,” “synonymous_variant”*; (iv) Variant Allele Frequency (AF) is >0.001 in at least one population; (v) Variant site is covered in ≥80% of the individuals in each seven populations; (vi) Variant is “PASS” quality in exome and genome datasets (if present in both); (vii) Variant site does not contain frequent alternative variants that could compromise statistical results of the biallelic HWE test (sum of AFs of all alternative variants seen at the same chromosomal position in the same population must be <0.001).

### Statistics and Measuring Deviations From HWE

The original code to measure statistical significance of variant deviation from HWE, developed by Wigginton et al. (2005), calculated *P* (two-sided) as the probability of observed sample plus the sum of all probabilities of more extreme cases. However, Graffelman and Moreno (2013) later showed that mid *P*, calculated by adding only half of the probability of observed sample to the sum of all probabilities of more extreme cases, was less conservative (i.e., mid *P* is always smaller than two-sided *P*) and showed better potential for testing deviations from HWE of rare variants. Therefore to create a python implementation of Graffelman and Moreno method, we modified Wigginton et al. code to return mid P. Variants deviating from HWE with mid *P* ≤ 0.05 were considered to be statically significant. For all other cases two-sided Fisher’s exact test was used (SciPy python package; Virtanen et al., 2019), and the results were reported as *P* and Fold Enrichment (FE), defined as the ratio of the two proportions. The code can be found at https://github.com/niab/hwe.

### Selecting Candidate Disease/Heterozygote Advantageous Variants

Variants that satisfied the following criteria were selected into a final dataset of candidate disease/heterozygote advantageous variants: (i) Variant AF is ≤0.05 in each of the ethnic populations [more common variants are classified as “benign” according to American College of Medical Genetics and Genomics (ACMG) guidelines; Richards et al., 2015, and are more likely to deviate from HWE due to genotyping errors; Karczewski et al., 2019b]; (ii) Variant has statistically significant (*P* ≤ 0.05) excess of heterozygotes in at least one ethnic population; (iii) Variant has excess of heterozygotes in each population (not required to be statically significant). This filter was added as variants with heterozygote excess in one ethnic population but not in the others, might be a result of gene flow; (iv) Variant is not located in a segmental duplication (Bailey et al., 2002) or tandem repeat region (Benson, 1999) (loci obtained from UCSC Genome Browser (Graffelman et al., 2017; Haeussler et al., 2019); (v) 50% of heterozygote variant carriers in the overall population has Allele Balance (AB), defined as the proportion of reads that support the minor allele^2^, between 0.4 and 0.55 (AB thresholds are justified in the Results section). After applying these filters the resulting dataset consisted of 299 variants located in 267 genes.

A recent investigation of the deviation from HWE of the CCR5-Δ32 allele in gnomAD showed that excess of heterozygotes can be caused by misclassification of homozygous individuals as heterozygous with high AB (Karczewski et al., 2019b). To minimize the number of false positive candidate disease/heterozygote advantageous variants, HWE statistics for them was recalculated considering heterozygous individuals with AB > 0.8 as homozygous, which is a more conservative than the 0.9 AB threshold used in the original study (Karczewski et al., 2019b). AB data was not available for each ethnic population, so an assumption was made that novel homozygous individuals were distributed among populations in the same proportions as heterozygotes. After excluding variants that were no longer deviating from HWE due to heterozygote excess, the final dataset consisted of 161 variants located in 149 genes (Supplementary Table S1). HWE statistics of these variants was recalculated using gnomAD v3 data (71,702 whole genome samples mapped to build GRCh38), which contained a larger AFR population (∼1.7 times larger; 21,042 individuals, all other populations were smaller than in gnomAD v2.1.1). Chromosome coordinates were mapped with LiftOver (Haeussler et al., 2019).

## Results

**FIGURE 1:**
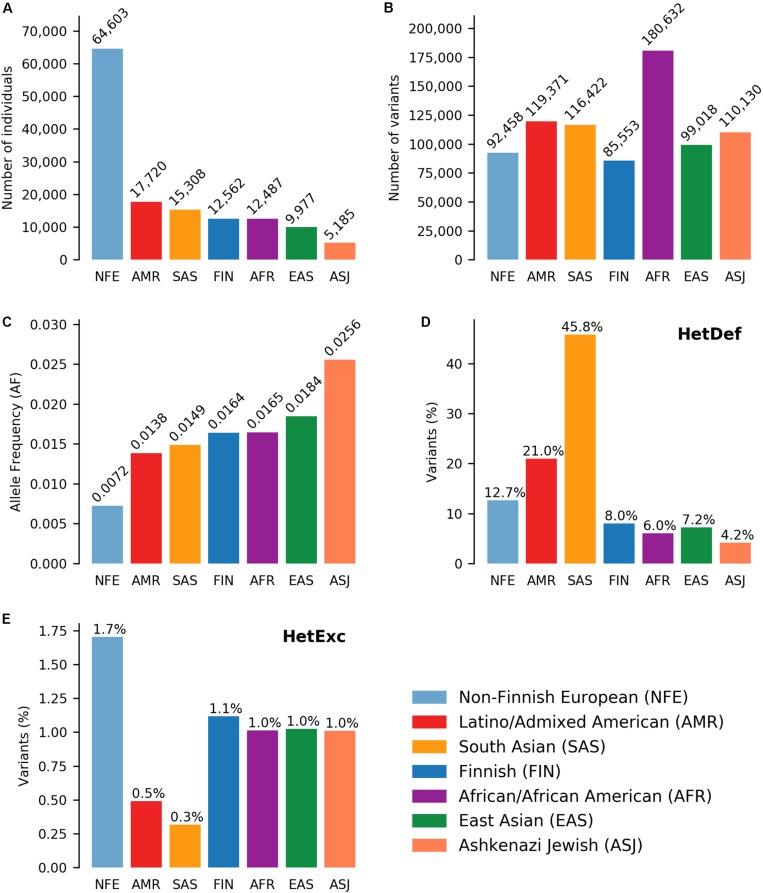
Deviations from Hardy-Weinberg Equilibrium (HWE) in 7 ethnic gnomAD populations. Number of **(A)** individuals and **(B)** variants in each population. **(C)** Minimum variant Allele Frequency (AF) required for statistically significant heterozygote excess according to HWE, in the absence of homozygous individuals in each population. Percentage of variants (raw numbers are shown in **B**) deviating from HWE due to **(D)** heterozygote deficiency or **(E)** heterozygote excess in each population.

After applying initial filters on variant data from seven ethnic populations (Figure 1A), the resulting dataset consisted of 382,506 unique variants (803,584 if counted in each population separately, Figure 1B) located in 16,871 genes. Exclusion of rare variants (AF < 0.001) from the analysis reduced the possible impact of population size (Figure 1A) on the number of variants analyzed (Figure 1B). For example, the Finnish (FIN) populations was ∼5 times smaller than the Non-Finnish European (NFE) population (12,562 and 64,603 individuals, respectively), but had a similar number of unique variants (85,553 and 92,458 variants, respectively). However, population size had a significant effect on the ability of the HWE test to detect Heterozygote Excess (HetExc) deviation of rare variants: the larger the population, the smaller the AF threshold after which statically significant HetExc can be reported. The minimal HetExc AF thresholds (i.e., assuming complete absence of homozygotes) are shown on Figure 1C, note the negative correlation with population sizes shown in Figure 1A.

**FIGURE 2:**
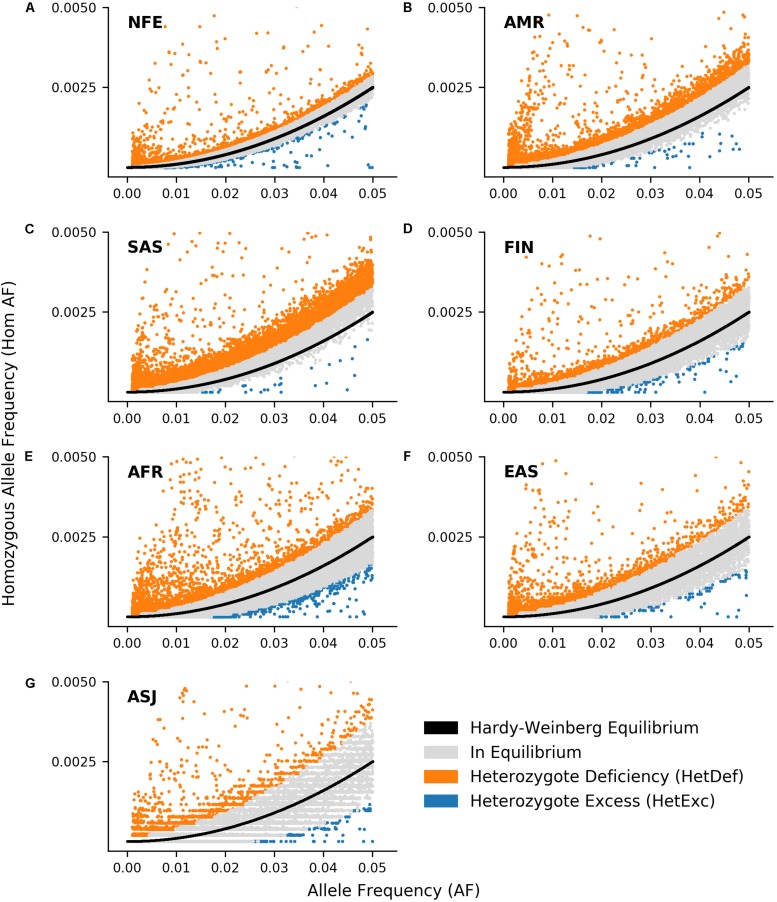
Comparison of observed ratio between variant Alllele Frequency (AF) and homozygous AF with expected ratio according to Hardy-Weinberg Equilibrium (HWE) in 7 ethnic gnomAD populations: Non-Finnish European (**A**, NFE), Latino/Admixed American (**B**, AMR), South Asian (**C**, SAS), Finnish (**D**, FIN), African/African American (**E**, AFR), East Asian (**F**, EAS), and Ashkenazi Jewish (**G**, ASJ). Black line represents expected ratio between AF and expected homozygous AF according to HWE. Variants where deviation from HWE are not significant (*P* > 0.05) are shown in gray, whereas those that deviate from HWE due to heterozygote deficiency or excess are shown in orange and blue respectively. Only variants with 0.001 ≤ AF ≤ 0.05 and homozygous AF ≤ 0.005 are shown.

Another factor that could affect detection of HetExc variants, was the degree to which HWE assumptions were satisfied in each population. For example the “random mating” assumption would be violated in populations with a high degree of consanguineous marriages or consisting of individuals from several countries, and would result in a higher proportion of variants deviating from HWE due to Heterozygote Deficiency (HetDef) (i.e., “Wahlund effect”). To some degree, all populations deviated more frequently from HWE due to HetDef than HetExc (Figures 1D,E). The largest proportion of HetDef variants were observed in South Asian (SAS) and Latino/Admixed American (AMR) populations, 45.8 and 21.0%, respectively. Consequently, these populations also had the lowest proportion of HetExc variants, 0.3 and 0.5%, respectively. The lowest proportion of HetDef variants was observed in the Ashkenazi Jewish (ASJ) population. However, even in this population, variants deviated from HWE due to HetDef ∼4 times more frequently than due to HetExc, 4.2 and 1.0%, respectively. Interestingly, the African/African American (AFR) population had the second lowest percentage of HetDef variants (6.0%), which outscored the FIN population (8.0%), considered as a homogeneous isolate. The largest proportion of HetExc variants was in the NFE population (1.7%, 1,574 variants), which had the smallest AF threshold for HetExc detection (AF = 0.0072, Figure 1C). Despite this, the AFR population still had the largest absolute number of HetExc variants (1,829). Therefore, overall population variant shift from HWE toward HetDef (i.e., the majority of the variants have higher than expected homozygous AF) decreased the number of statistically significant HetExc variants (especially in SAS and AMR), which can also be seen in Figure 2 for relatively rare variants (AF < 0.1).

This initial analysis has been performed on variants that remained following the gnomAD sequence quality filtering process and might therefore be assumed to be real. However, variant databases are known to contain errors that could give a significant HetExc signal. To explore this we developed a set of more stringent filters. In particular, variant properties that could produce a false positive HetExc signal were investigated. For this analysis, variants present in multiple populations were counted once, and variants with AF > 0.05 in at least one population were excluded. At this stage, only variants that had an excess of heterozygotes in all populations, and were statistically significant in at least one population were classified as HetExc.

**FIGURE 3:**
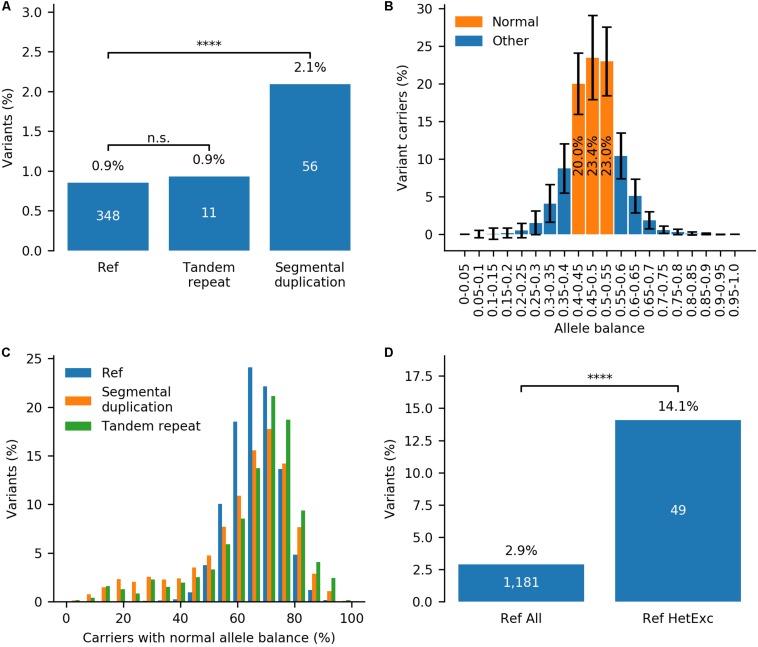
Impact of tandem repeats, segmental duplications and allele balance on the probability of variant deviation from Hardy-Weinberg Equilibrium (HWE) due to heterozygote excess (HetExc). **(A)** Percentage of variants deviating from HWE due to HetExc that are located in tandem repeat, segmental duplication regions or the reference (“Ref,” all other regions) group. **(B)** Distribution of Allele Balance (AB) between variant carriers in variants from “Ref” group (error bars indicate standard deviation). For each variant these statistics are aggregated into a single metric that represents cumulative percentage of Variant Carriers with Normal (0.4–0.55) Allele Balance (VCNAB, e.g., 20.0% + 23.4% + 23.0% = 66.4%). **(C)** Distribution of variants with various VCNAB percentages in “Segmental duplication”, “Tandem repeat” and “Ref” groups. **(D)** Percentage of variants with VCNAB < 50% in the whole “Ref” group and a subset of variants with statistically significant excess of heterozygotes in “Ref” group. ^****^Indicates statistical significance of *p* ≤ 0.0001.

Firstly, to investigate the correlation between HetExc and chromosomal regions prone to sequencing errors, variants were divided into three groups: (i) “segmental duplication” (2,676), (ii) “tandem repeat” (1,182), and (iii) all others named “Ref” (40,801). HetExc variants were significantly more frequent in the “segmental duplication” (FE = ∼2.5, *P* = 2.0E-08) group than in the “Ref” group, whereas the proportion of HetExc variants in the “tandem repeat” and “Ref” groups were almost the same (Figure 3A and Supplementary Table S2). Therefore, HetExc of variants located in segmental duplications might be a result of genotyping errors.

Secondly, to investigate the correlation between HetExc and Allele Balance (AB), which is a known indicator of systematic genotyping errors (Muyas et al., 2019), the AB profile of an average gnomAD variant was required. In gnomAD, variant AB data is stored as a number of variant carriers (converted to percentages here) in 20 AB groupings (from 0 to 1, 0.05 group size). Figure 3B shows the distribution of AB between variant carriers in variants from the “Ref” group. For an average variant, the majority of variant carriers (66.4%) had an AB between 0.4 and 0.55 and were named “Normal” here, because it was close to the expected normal 0.5 ratio for heterozygous variants. To aggregate variant AB data from 20 groups into a single numeric metric, it was measured as percentage of Variant Carriers with “Normal” Allele Balance (VCNAB), calculated as the number of heterozygote variant carriers with AB 0.4–0.55 divided by the total number of heterozygote variant carriers. Both “segmental duplication” and “tandem repeat” groups had more variants with high and low VCNAB [80% Confidence Interval (CI) = 33.9–79.6% and 41.9–81.4%, respectively] than the “Ref” group (80% CI = 55.6–77.0%), which indicates that variants in these regions are more prone to genotyping errors and were excluded from further analysis (Figure 3C). The minimal VCNAB threshold for “PASS” quality variants was defined by a lower bound fraction of 95% CI calculated for variants from the “Ref” group (CI = 49.3–82.2%), rounded to 50% (i.e., half of the variant carriers must have AB in the range 0.4–0.55). Only 2.9% of variants in the “Ref” group would not pass this filter, but the fail rate among HetExc variants would be ∼4.9 times higher (*P* = 1.9E-17, Figure 3D and Supplementary Table S3). Therefore, variants with low AB (VCNAB < 50%) might be enriched with genotyping errors and were also excluded from further analysis.

**FIGURE 4:**
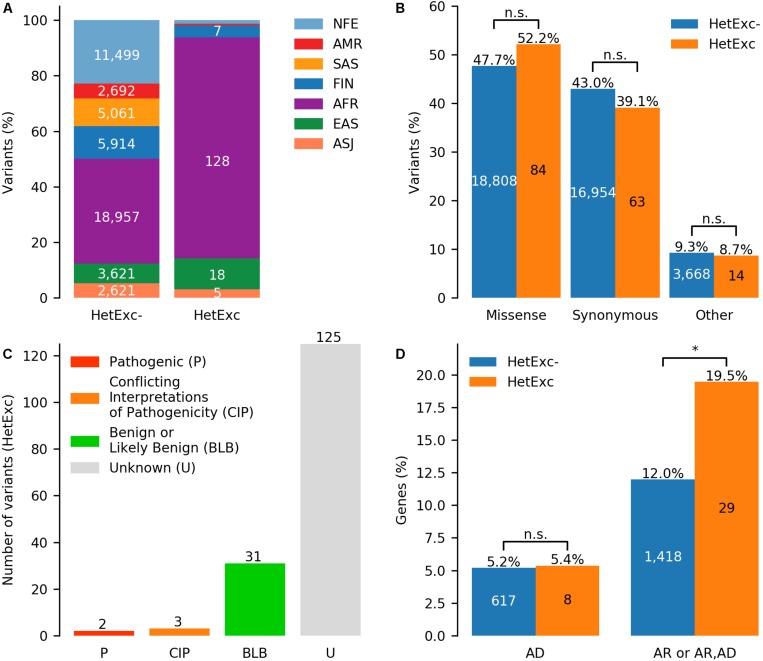
Potential recessive disease causing variants identified by deviation from Hardy-Weinberg Equilibrium (HWE) due to excess of heterozygotes (HetExc). **(A)** Distribution of variants deviating and not deviating from HWE due to excess of heterozygotes (HetExc and HetExc-, respectively) in 7 ethnic gnomAD populations. **(B)** Proportions of missense, synonymous and other protein coding variants in HetExc and HetExc- datasets. **(C)** ClinVar status (e.g., pathogenic/benign) of HetExc variants **(D)** Known disease associated genes with at least one variant in HetExc and HetExc- datasets grouped by inheritance pattern: autosomal dominant (AD), autosomal recessive (AR) or both. ^∗^Indicates statistical significance of *p* ≤ 0.05.

Finally, HWE statistics for HetExc variants that were not located in segmental duplication or tandem repeat regions and had VCNAB ≥ 50% (299 variants in 267 genes) were recalculated considering heterozygous individuals with AB > 0.8 as homozygous. 161 variants in 149 genes that were still HetExc according to the updated HWE statistics were selected as candidate recessive disease causing genes (Supplementary Table S1). These HetExc variants were then compared with a group of variants that survived the same filtering process, but did not have an excess of heterozygotes (HetExc-). The HetExc- and HetExc groups consisted of 39,430 and 161 variants (50,365 and 161 if counted in seven ethnic populations separately, Figure 4A) in 11,842 and 149 genes, respectively. Most of the HetExc variants were present in African/African American populations (128/161, ∼79.5%), which was significantly more than expected (FE = 1.7, *P* = 3.0E-05) based on the proportion in the HetExc- group (18,957/39,430), whereas all other populations had significantly less than expected HetExc variants (*P* ≤ 0.001) except EAS and ASJ (Supplementary Table S4). Both HetExc- and HetExc groups contained a similar proportion of missense and synonymous variants (Figure 4B and Supplementary Table S5).

To determine which of the HetExc candidate recessive disease causing variants were already known, their clinical significance in the disease variant database (ClinVar; Landrum et al., 2016) was analyzed. The majority of HetExc variants (125/161, 77.6%) were not present in ClinVar, whereas the majority of those that were present in ClinVar (31/36, 86.1%) had a “Benign” or “Likely benign” status (Figure 4C). The only two variants with “Pathogenic” status were c.20A > T (rs334) in *HBB* (causes recessive sickle cell disease MIM:603903; carriers are protection from malaria, MIM:611162) and c.1521_1523delCTT (rs1801178) in *CFTR* [causes recessive cystic fibrosis disease, MIM:219700; hypothesized to be protective from cholera (Rodman and Zamudio, 1991) or tuberculosis (Bosch et al., 2017)]. However, genes with at least one HetExc variant were significantly more frequently associated with known autosomal recessive (AR) diseases than genes containing only HetExc- variants (FE = ∼1.6, *P* = ∼0.02, Figure 4D and Supplementary Table S6). HetExc variant enrichment in known AR genes adds evidence that some of the selected variants might deviate from HWE due to natural selection and could have some disease association. However, only seven (∼5.5%) of these variants were also HetExc in gnomAD v3: c.20A > T (rs334) in *HBB*, c.7210G > C (rs61292917) in *CHD6*, c.3118A > G (rs34805848) in *TRIP11*, c.9540G > A (rs62642506) in *HSPG2*, c.1691G > A (rs751887) in *MMP24*, c.626C > T (rs35157957) in *RPUSD4* and c.441C > T (rs73887968) in *TCF20*. Note that 3/7 (∼42.9%; *HBB*, *TRIP11*, and *HSPG2*) genes are associated with known recessive diseases (Supplementary Table S1). Since gnomAD v3 mostly consisted of individuals that were not present in gnomAD v2.1.1, this adds evidence that these seven variants are not deviating from HWE by chance.

## Discussion

Analysis of deviations from the HWE on a large genomic dataset has shown that all populations, but especially South Asian (SAS) and Latino/Ad-mixed American (AMR), were more frequently deviating due to heterozygote deficiency (HetDef) than heterozygote excess (HetExc). A higher rate of HetDef variants in SAS and AMR populations is in line with previous reports (Gazal et al., 2015; Chen et al., 2017), possibly due to the large number of consanguineous marriages in these regions [e.g., 38% of SAS population in the Exome Aggregation Consortium (ExAC); Lek et al., 2016]. However, our findings that HetDef is a major cause of deviations from HWE in all populations is contrary to previous studies (Chen et al., 2017; Graffelman et al., 2017, which used more strict *P* thresholds (0.001 and 0.0001, respectively) and reported that deviations from HWE were more frequently observed due to HetExc. However, previous studies focused on error detection in older and smaller datasets, some of which were corrected in gnomAD. Graffelman et al. analyzed 104 Japanese individuals in the 1000 Genomes database (The 1000 Genomes Project Consortium, 2015), where the minimal statistically significant HetExc AF threshold (0 homozygous and *P* < 0.001) was ∼0.23 (Graffelman et al., 2017). Only 11/382,506 variants analyzed in our study were that frequent and had no homozygous individuals reported, nine of which were located in segmental duplication or tandem repeat regions. We observed a higher rate of HetExc variants in these regions, as well as those that had low allele balance, which correlates with previous work (Graffelman et al., 2017; Muyas et al., 2019). Chen et al. (2017) analyzed “open reading frame” genes and selected only one variant per gene where AF was closer to 0.50 (584 variants in total) in ExAC (60,706 individuals). However, this approach resulted in the exclusion of rare variants that were analyzed in this study and might be more affected by the Wahlund effect (i.e., more likely to be HetDef). Moreover, some of the HetExc variants detected in previous studies were marked as non-pass quality or were no longer HetExc in gnomAD, possibly due to differences in variant filtering and genotype calling procedures. For example, c.1801C > T (rs1778112) in *PDE4DIP* was present in the heterozygous state in ∼91% of individuals in the 1000 genomes database, but was never observed as homozygous and was assigned “non-pass” quality in gnomAD. Another example, the *BRSK2* variant c.551 + 6delG (rs61002819) was HetExc in ExAC (*P* = 1.9E-15), but not in gnomAD (*P* = 0.13). Therefore, a higher rate of HetDef variants in our study could be explained by a larger population size and a different variant dataset, as well as improvements in variant filtering and genotype calling procedures.

Analysis of HetExc variants (Supplementary Table S1), selected as recessive disease causing candidates, led to somewhat contradictory results, which should be interpreted with caution. Enrichment of HetExc variants in the African/African American (AFR) population was unexpected, and might indicate more extensive natural selection or be a sign of systematic genotype errors in this population. Enrichment of HetExc variants (32/161) in genes associated with known autosomal recessive diseases supports the hypothesis that some of these variants could be causing recessive diseases, whereas the presence of a large proportion of synonymous variants (11/32) and the assigned “Benign” or “Likely benign” status of the majority of the known variants (21/32 in CinVar, 17/21 were “Benign” or “Likely benign”) in this group provides evidence against it. Moreover, despite applying our extensive filtering strategies, many of the HetExc variants might still be deviating from HWE due to genotype errors or by chance due to insufficient population size. The latter might be an explanation for some AFR variants that were HetExc in gnomAD v2.1.1, but not in the new v3 release, which had a larger AFR population. However, the c.1521_1523delCTT (rs1801178) variant in *CFTR* also was not HetExc in gnomAD v3 and was observed as homozygous in 4/32,299 NFE individuals (and 2 heterozygotes with AB > 0.8), whereas in v2.1.1 only 1/64,603 NFE individuals was homozygous. Therefore, the difference between the number of homozygote in gnomAD v2.1.1 and v3 might also be explained by other factors, such as differences between genotype calling procedures for exome and genome data.

Nevertheless, the presence of known pathogenic and heterozygote advantageous variants such as *HBB* c.20A > T and *CFTR* c.1521_1523delCTT suggests that some of the other 161 HetExc variants might also be functionally significant. Especially, the *CHD6* gene variant c.7210G > C (rs61292917), which was HetExc in both versions of the gnomAD database and was predicted to be deleterious by *in silico* tools (SIFT = 0; Ng and Henikoff, 2003; PolyPhen-2 = 0.961; Adzhubei et al., 2013). Moreover, it was more frequently (FE = 5.21, *P* = 1.19E-04) seen in African than African American populations in the 1000 genomes database (Supplementary Table S7), similar to the c.20A > T variant in *HBB* (FE = 3.42, *p*-value = 1.49E-05), which suggests that these variants might be under purifying selection in populations that moved out of Africa (i.e., they might be disease causing, but advantageous only in Africa, which is known in the case of *HBB* c.20A > T). Although *CHD6* is not yet linked with any disease, it is known to act as transcriptional repressor of different viruses including influenza and papiloma virus (Alfonso et al., 2011, 2013). Interestingly, c.7210G > C has a much lower AF in the African population (AF = 0.066), than c.20A > T (AF = 0.120) in the 1000 genomes database. Considering *CHD6* is extremely intolerant to variation (GeVIR = 5.30%; Abramovs et al., 2020, LOEUF = 0.07; Karczewski et al., 2019a), c.7210G > C is more enriched in the African population compared with c.20A > T (i.e., possible due to stronger purifying selection), this suggests that c.7210G > C might be disease causing even in the heterozygous state.

Our study highlighted that the ability of HWE to detect candidate recessive disease causing variants is mainly limited by both the quality of genotype calls and the size of available exome/genome variant data, whereas absence of information about sub-populations (e.g., Africans and African Americans) and a high level of inbreeding (e.g., SAS) could reduce sensitivity, but not precision, of the approach in certain populations. We anticipate that improvements in sequencing technologies and variant filtering software should reduce the number of false positive HetExc variants in the future. In fact, false positive HetExc variants that survived our strict quality filters, might aid the development of more efficient sequencing filtering strategies by helping to understand new patterns of genotype errors. The size of the largest population analyzed in this study (NFE = 64,603 individuals) allowed us to detect statistically significant HetExc only amongst variants with AF ≥ ∼0.0072 (∼33% of 61,077 variants with AF = 0.001–0.05). Consequently, some common recessive disease causing variants were missed even if homozygous individuals were completely absent in the population. For example, HetExc of the c.448G > C (rs1800546) variant in *ALDOB* (causes recessive hereditary fructose intolerance) was not statistical significant (*P* = ∼0.3), despite being observed in the heterozygous state in 627 NFE individuals (AF = ∼0.005). As the number of sequenced exomes and genomes is rapidly growing, this problem may soon be addressed. Indeed, the United Kingdom National Health Service is planning to sequence 1 million genomes in the next 4 years with a wider ambition to increase this number to 5 million^3^. If the NFE population was 1 million, then the AF threshold would drop to ∼0.0018 (∼73% of 61,077 variants with AF = 0.001–0.01), whereas with 5 million individuals it would be possible to detect statistically significant HetExc in all variants with AF ≥ ∼0.0008. Therefore, it might be possible to use HWE strategies to detect rare recessive disease causing variants in the near future.

## Conclusion

In this study, we explored the use of HWE to identify potential recessive disease causing variants in a large mainly healthy population database by developing a bespoke filtering strategy to detect variants where an excess of heterozygotes in a population could be a result of natural selection. Overall, this approach showed potential, especially for the AFR population, successfully identifying some variants in recessive diseases that are known to be heterozygote advantageous, and providing novel candidates for further investigation. A natural progression of this work would be validation of genotype calls of HetExc variants to understand possible causes of genotype errors and analysis of the biological effect of true positive HetExc variants to determine their potential health implications. We also anticipate that this approach will become more robust in the future as the size and quality of available genomic data increases.

## Data Availability Statement

Publicly available datasets were analyzed in this study. This data can be found at the following links: https://console.cloud.google.com/storage/browser/gnomad-public/release/2.1.1/; ftp://ftp.1000genomes.ebi.ac.uk/vol1/ftp/release/20130502/.

## Author Contributions

NA, MT, and AB conceived and designed the research. NA executed the analysis. NA and MT performed the primary writing. MT and AB supervised all aspects of the research, reviewed, and edited the manuscript.

## Conflict of Interest

The authors declare that the research was conducted in the absence of any commercial or financial relationships that could be construed as a potential conflict of interest.

## Footnotes

1 https://gnomad.broadinstitute.org/api

2 https://gatk.broadinstitute.org/hc/en-us/articles/360036479772-FilterVcf-Picard-

3 https://www.gov.uk/government/news/matt-hancock-announces-ambition-to-map-5-million-genomes

## Supplementary Material

TABLE S1 Dataset of variants deviating from Hardy-Weinberg Equilibrium due to heterozygote excess (HetExc).

TABLE S2 Statistical comparison of variants deviating from HWE due to HetExc that are located in segmental duplication **(A)** or tandem repeat **(B)** regions with the reference (Ref) group (i.e., all other regions except segmental duplications and tandem repeats).

TABLE S3 Statistical comparison of variants with Variant Carriers with “Normal” Allele Balance VCNAB < 50% in the whole Ref group and a subset of variants with statistically significant excess of heterozygotes (HetExc) in the Ref group.

TABLE S4 Statistical comparison of proportions of variants deviating and not deviating from HWE due to excess of heterozygotes (HetExc and HetExc-, respectively) in 7 ethnic gnomAD populations (**A–G**).

TABLE S5 Statistical comparison of missense **(A)**, synonymous **(B)**, and other **(C)** variant proportions in HetExc and HetExc- datasets.

TABLE S6 Statistical comparison of proportions of “AD” **(A)**, “AR or AR, AD” **(B)** and all genes with at least one variant in HetExc and HetExc- datasets.

TABLE S7 Statistical comparison of Allele Frequencies of heterozygote excess (HetExc) variants in *HBB* and *CHD6* genes between African and African American population in the 1000 Genomes database.
